# Continuous passive motion not affect the knee motion and the surgical wound aspect after total knee arthroplasty

**DOI:** 10.1186/s13018-022-02916-w

**Published:** 2022-01-15

**Authors:** Sergi Gil-González, Ricardo Andrés Barja-Rodríguez, Antoni López-Pujol, Hussein Berjaoui, Jose Enrique Fernández-Bengoa, Juan Ignacio Erquicia, Joan Leal-Blanquet, Xavier Pelfort

**Affiliations:** 1 Hospital Universitari Igualada, Consorci Sanitari de L’Anoia, Av. Catalunya, 11, 08700 Igualada, Barcelona Spain; 2 Hospital Universitari Parc Taulí, Parc Taulí, 1, 08208 Sabadell, Barcelona Spain

**Keywords:** Total knee arthroplasty, Continuous passive motion, Knee motion, Surgical wound aspect, Pain

## Abstract

**Background:**

This study aimed to assess whether use of continuous passive motion (CPM) could improve range of motion in patients after total knee arthroplasty (TKA), if it could affect the surgical wound aspect (SWA) and if it could influence on pain management after TKA.

**Methods:**

We randomized 210 patients in two groups, 102 patients in the CPM group, who received a standard rehabilitation protocol together with CPM application; and 108 patients in the no-CPM group, without CPM. Variables as knee motion (flexion, extension, range of motion) and pain were measured before surgery, on the 1st, 2nd and 3rd postoperative day, and in the 2nd, 6th, 12th and 24th postoperative weeks following TKA. The SWA was determined by the “surgical wound aspect score” (SWAS) in the next 48 h after surgery. This scale analyzes swelling, erythema, hematoma, blood drainage and blisters.

**Results:**

There was an improvement in the knee motion over the course of follow-up in both groups, without significant difference in flexion parameter. We found no significant differences in the total score of SWA, except for hematoma, with less severity in the CPM group. Furthermore, we found no differences in the others SWAS parameters and pain.

**Conclusions:**

The application of CPM does not provide benefit to our patients undergoing TKA in terms of either improved flexion mobility or decreased pain. No relationship was found between the use of CPM and the global score of SWA following a TKA, except for a decrease in hematoma appearance.

## Introduction

Knee osteoarthritis (OA) is a common disorder which generate severe pain, deformity and reduced knee mobility [1]. Total knee arthroplasty (TKA) is one of the best methods to treat knee OA, reducing pain, improving range of motion (ROM) and recovery of knee function [2, 3]. Since the 1970s, continuous passive motion (CPM) has been used to improve knee mobility and reduce stiffness after TKA, being part of the rapid postoperative recovery programs [4–6]. This intervention is provided by a machine that performs repetitive passive motion. The main benefits of CPM described in the literature are: improvement in the range of motion, decreased pain and reduced swelling, improvement in local circulation and reduced need for manipulation under anesthesia [7]. Nevertheless multiple studies show that the application of CPM does not have long-term advantages [4, 5, 8–14]. Moreover, some studies found no differences comparing the addition of CPM in these rehabilitation programs after TKA, versus patients who only received standard physical therapy alone [15–18]. Concerning short-term benefits, in the last two decades the controversy has grown. Some studies suggest that patients who have received CPM after TKA have faster recovery in ROM [4, 9, 11, 14, 17, 19–22], less stiffness [22], less pain [23] and lower incidence of thrombophlebitis [21]. On the other hand, some studies find an increase in swelling [5, 13] and higher levels of pain [13]. Few articles analyze the relationship of wound healing and the use of CPM, being this association a fairly unknown topic [24]. Despite all the contradictory information, CPM is widely used in hospitals around the world as part of the standard postoperative management protocol for TKA [7].

The objectives of this study are to assess whether the use of CPM can improve range of motion in patients after TKA in comparison to a conventional self-assisted rehabilitation program without CPM, correlation between their use and the surgical wound aspect (SWA) and the influence in the level of pain.

## Materials and methods

### Participants

Between January and December 2018, a prospective, randomized controlled trial was performed. All patients over 50 years of age and implanted with a primary TKA due to knee OA, were asked to enrol in our study. Patients with a high degree of deformity in the mechanical axis (over 15° in varus or valgus deformity) or contracture knee flexion (> 20°), inflammatory arthropathies, previous surgeries in the same knee (except simple arthroscopies), were excluded. All patients included signed an informed consent. The hospital’s ethical committee approved the study (PR180/19). This study was performed in line with the principles of the Declaration of Helsinki.

### TKA procedure

All the surgical procedures were carried by the same four senior consultants. All of them used two types of posterior stabilized cemented TKA: Stryker^®^ Triathlon (Stryker, Mahwah, NJ, USA) or Surgival^®^ Genutech (Surgival, Valencia, España) employing a medial parapatellar surgical approach. Intradural anaesthesia was used for all patients without regional nerve blocks and a pneumatic tourniquet was inflated to 300 mmHg pressure before the incision and deflated at the end of surgery after skin closure. Local infiltration with 10–20 cc of Ropivacaine or Bupivacaine was administrated. A suction drain was introduced before wound closure and removed on the first postoperative day. Wound closure was made with simple stiches of coated vicryl^®^ suture (polyglactin 910, Ethicon, Inc.) on subcutaneous tissue and skin with staples. Wound dressing was made with two gauzes and a soft bandage, removed at same time that suction drain, at the 24th postoperative hours. Patients received low weight heparin as venous thromboembolism prophylaxis for 30 days. Multimodal pain management protocol was applied in all patients.

### Randomization and application of the treatment

**Fig. 1:**
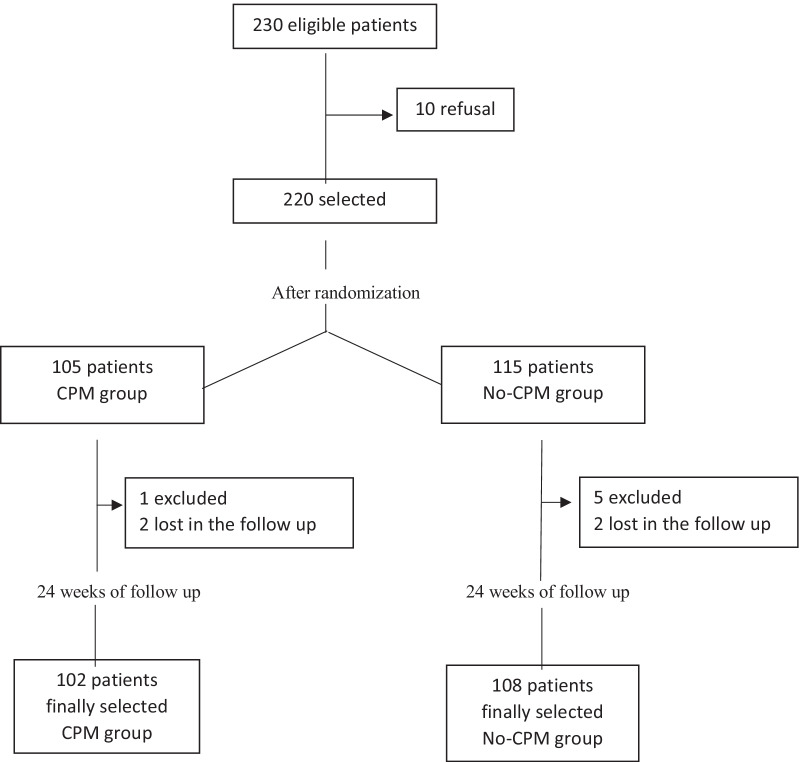
Flowchart of the participants recruitment

Of the 230 eligible patients, 220 participants were assigned to each group according to a randomization list. (Fig. 1). The participants of the same group were cited in the same week to accomplish the treatment. That means that all the patients were surrounded with other patients that had the same rehabilitation program, in order to avoid possible information biases. One hundred and five patients were included in the CPM group, and they received a standardized rehabilitation program (SRP) with CPM. The others 115 patients received the same SRP without CPM (no-CPM group). The appointments were: (1) the same day of surgery, treatment of SRP was applied and it consist on assisted physical exercises carried out by a professional trained physiotherapist; (2) the following appointments were three times per day with at least 1 h on each occasion, and involving 20 repetitions for every exercise, including ankle mobility, active isometric contraction of the quadriceps, straight leg raises, quad sets and physiotherapist-assisted knee mobility exercises. In the CPM group, all patients started the CPM therapy the day of surgery and during two hours of therapy per three sessions at day until discharge. The degree of flexion was adjusted by the physiotherapist according to patient tolerance and progression at each session. Following discharge, a physiotherapist continued rehabilitation and knee mobility exercises without CPM at home for all patients in both groups, for one hour every day for the following ten consecutive days.

### Data collection

The main variable was collected using double-blind by the rehabilitation consultant and the senior surgeon. Only physiotherapist knows the application or not of CPM. Knee ROM was measured using a long digital goniometer before surgery, on the 1st, 2nd and 3rd postoperative day, and in the 2nd, 6th, 12th and 24th postoperative weeks following TKA. The measurement was carried out by the rehabilitation consultant after the rehabilitation session of each day, and in the different visits of follow-up. The aspect of the surgical wound was assessed by the “surgical wound aspect score” (SWAS) in the next 48 h after surgery by the senior surgeon. This scale analyzes five parameters of wound characteristic: swelling, erythema, hematoma, blood drainage and blisters, obtaining a value from 0 to 2 points for each parameter depending on the severity of the injury and resulting a total score from 0 to 10 (being 10 the worst condition of the wound) [25]. Postoperative pain was measured by a rehabilitation consultant using the visual analog scale (VAS) (values from 0 to 10), prior surgery, on the 1st, 2nd and 3rd postoperative day, and in the 2nd, 6th, 12th and 24th postoperative week following TKA.

### Statistical analysis

Descriptive results were calculated as means values and its standard deviations for continuous variables and a descriptive of the frequencies and its corresponding percentages for categorical variables. Differences in clinical characteristics and outcomes between study and control group were assessed using Mann–Whitney *U* tests for continuous variables and chi-squared and Fisher’s exact tests for categorical variables. In our statistical analyses, we accepted an alpha risk of 0.05 and a beta risk of 0.2 in a two-sided test. Anticipating a 20% drop-out rate, we aimed to recruit 187 consenting patients to achieve sufficient power. All analyses were performed based on intention-to-treat. All analyses were performed using IBM SPSS Statistics.

## Results

The randomization procedure created demographically similar groups, without differences in preoperative parameters like age, gender, mobility (extension, flexion, ROM) or pain (Table 1). The mean age of the patients was 74.23 (range: 55–87 years), with a non-statistical significant higher percentage of females (*n* = 137; 65.2%) compared to males (*p* = 0.895).

**Table 1:** Patient demographic and preoperative data

Variable	CPM group	No-CPM group	*P* Value
	(*n* = 105)	(*n* = 115)	
Age (years)	74.23 (6.79)	73.33 (6.9)	0.347
Gender (F:M)	67:38	70:45	0.895
Pre-op mobility (°)			
Extension	3.11 (4,29)	3.53 (5.63)	0.540
Flexion	110.48 (11.92)	109.21 (12.32)	0.445
Full ROM	107.38 (13.28)	105.67 (15.43)	0.393
Pre-op VAS	4.4 (2.4)	3.9 (2.4)	0.204

Values shown are mean (SD) *p* value significant at 0.05

In Fig. 1, we can observe that six patients were excluded from analysis because of postoperative complications that prevented application of the rehabilitation protocol. There were two patients with intraoperative fractures, two patients with deep infections that required two-stage revision, one with patellar tendon rupture and one that death during the follow-up. In addition, information from four patients who not completed follow-up, two in each group, was also excluded from the database.

**Fig. 2:**
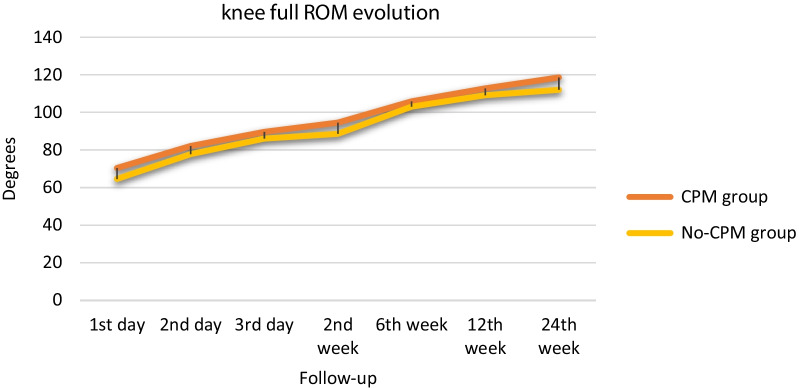
Evolution of knee full ROM trough follow-up in CPM and no-CPM groups

**Fig. 3:**
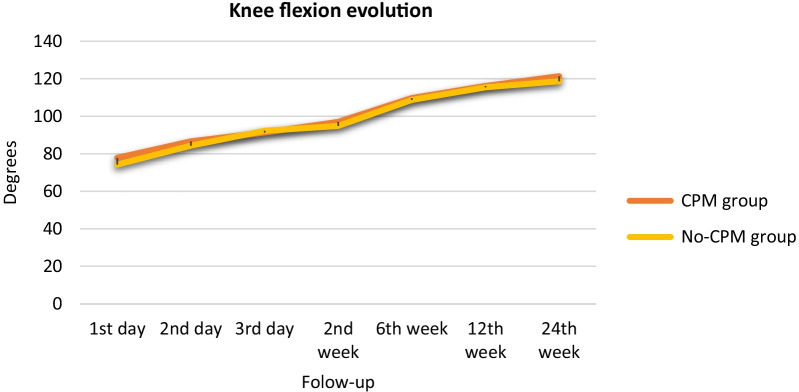
Evolution of knee flexion trough follow-up in CPM and no-CPM groups

Results comparing knee mobility are shown in Table 2. We found an improvement of flexion, extension and ROM, in both groups along the process between admission and discharge and over the course of follow-up (*p* < 0.010). ROM improved throughout the follow-up in both groups after the surgery (Fig. 2). However, the improvement in the CPM group was greater than the no-CPM group through all follow-up, being significant in the first and second postoperative day and in the second postoperative week of follow-up. At the 24th postoperative week, we observed a persistent improvement in ROM in the CPM group (6° greater in with respect no-CPM group), although this difference was not significant (*p* = 0.056). The most relevant parameter in the application of CPM was highlighting flexion. At the time of discharge, both groups again had similar results, with a mean flexion of 91.38° (SD 9.4) in the CPM group and 92.23° (SD 10.06) in the no-CPM group (*p* = 0.769). There were no significant differences between groups at any of the time points evaluated (Table 2). At the last follow-up, 24th postoperative week, mean flexion in the CPM group was 121.39° (SD 8.92) and 118.73° (SD 9.92) in the no-CPM group (*p* = 0.066) (Fig. 3). Concerning the extension, we did not observe any significant difference between groups. No patient had knee stiffness that required any surgical procedure or forced mobilization.

**Table 2:** Comparison of postoperative knee mobility in degrees in CPM and no-CPM groups

Follow-up	CPM group	No-CPM group	*P* value
Day 1			
Extension	7.48 (8.51)	9.14 (8.60)	0.164
Flexion	77.83 (13.32)	74.27 (16.05)	0.083
Full ROM	70.34 (13.62)	64.53 (17.04)	0.007
Day 2			
Extension	4.52 (5.93)	5.83 (6.57)	0.130
Flexion	86.59 (11.16)	84.33 (11.90)	0.158
Full ROM	82.07 (11.93)	77.76 (15.01)	0.023
Day 3			
Extension	1.11 (3.29)	2.7 (5.33)	0.064
Flexion	91.38 (9.40)	92.23 (10.06)	0.769
Full ROM	89.65 (13.75)	86.08 (15.49)	0.088
Week 2			
Extension	0.84 (3.19)	1.90 (4.99)	0.113
Flexion	97.01 (10.40)	94.89 (12.10)	0.180
Full ROM	94.57 (11.34)	88.62 (20.20)	0.010
Week 6			
Extension	0.31 (1.65)	0.54 (2.42)	0.484
Flexion	109.70 (10.35)	108.72 (11.84)	0.530
Full ROM	105.91 (18.61)	103.08 (21.90)	0.315
Week 12			
Extension	1.02 (2.74)	2.05 (4.16)	0.060
Flexion	116.17 (9.19)	115.44 (10.77)	0.603
Full ROM	112.89 (18.71)	109.19 (24.75)	0.225
Week 24			
Extension	0.65 (2.51)	1.23 (3.43)	0.175
Flexion	121.39 (8.92)	118.73 (9.92)	0.066
Full ROM	118.73 (19.59)	112.07 (27.08)	0.056

Values shown are mean (SD) *p* value significant at 0.05

Regarding the surgical wound, we did not find any difference in the total score of SWAS between CPM or in no-CPM groups (*p* = 0.289) (Table 3). However, when we analyzed the individual parameters of the SWAS, we found significantly fewer patients with a worse hematoma in the CPM group compared to the no-CPM group (*p* = 0.028). Despite this, any patient required surgical drainage or had skin necrosis due to the hematoma.

**Table 3:** Distribution of patients relative to the SWAS and comparison of CPM and no-CPM groups

SWAS	CPM group	No-CPM group	*P* value
Swelling			0.157
0	27 (26.5%)	17 (15.8%)	
1	57 (55.9%)	70 (64.8%)	
2	18 (17.6%)	21 (19.4%)	
Erythema			0.463
0	95 (93.1%)	97 (89.8%)	
1	7 (6.9%)	11 (10.2%)	
2	0	0	
Hematoma			0.028
0	58 (56.9%)	44 (40.8%)	
1	36 (35.3%)	46 (42.6%)	
2	8 (7.8%)	18 (16.6%)	
Drainage			0.558
0	68 (66.7%)	73 (67.6%)	
1	28 (27.5%)	25 (23.2%)	
2	6 (5.9%)	10 (9.2%)	
Blisters			0.175
0	98 (96.1%)	98 (90.7%)	
1	2 (2%)	8 (7.4%)	
2	2 (2%)	2 (1.9%)	
Total			0.289
0	11 (10.8%)	5 (4.6%)	
1	36 (35.3%)	29 (26.9%)	
2	25 (24.5%)	34 (31.5%)	
3	12 (11.8%)	16 (14.8%)	
4	12 (11.8%)	11 (10.2%)	
5	3 (2.9%)	7 (6.5%)	
6	2 (2%)	4 (3.7%)	
7	–	2 (1.9%)	
8	1 (1%)	–	
9	–	–	
10	–	–	

SWAS, Surgical wound aspect scale

Values shown are number and percentage of patients for each parameter and score level. *P* value significant at 0.05

From surgery, pain levels experienced by the patients were similar, with a comparable decrease over time in both groups (Table 4).

**Table 4:** Comparison of postoperative pain in CPM and no-CPM groups

VAS	CPM group	No-CPM group	*P* Value
Day 1	6.4 (1.9)	6.5 (2.0)	0.773
Day 2	5.6 (1.8)	5.8 (1.8)	0.497
Day 3	4.9 (1.7)	5.0 (1.7)	0.831
Week 2	4.4 (1.9)	4.4 (1.8)	0.997
Week 6	2.3 (2.0)	2.4 (2.0)	0.545
Week 12	1.1 (1.6)	1.1 (1.9)	0.914
Week 24	0.5 (1.1)	0.6 (1.3)	0.598

VAS, Visual analog scale (0–10)

Values shown are mean (SD) *p* value significant at 0.05

## Discussion

The main findings of our study are the lack of benefit to the knee flexion, surgical wound healing and reduction pain of the use of CPM. Our results are in agreement with many recent studies [20, 26] where they found no improvement on knee flexion due to application of CPM. We found no significant difference between groups in extension or flexion. However, we found a suggestion of higher degrees value of ROM in CPM group than in no-CPM group, observing around five degrees of difference in favor of the CPM group, especially on the 1st postoperative day or 2nd postoperative week, however we did not consider that difference could represent a significant clinical improvement. These results are in line with those reported in the Cochrane review [20], with a difference of two degrees to six in favor of CPM application in knee flexion between CPM and non-CPM group. However, some studies found different results to ours. McInnes et al. [27] or Lenssen et al. [28] described an increment in active flexion on the earlier postoperative days, in patients who received CPM, but not long-term benefit. Liao et al. [29] obtain an improvement on ROM at 3rd and 6th month follow-up with an early application of CPM with initial high flexion angle and rapid progress.

We observed a similar improvement from the 1st postoperative day to 24th postoperative week of follow-up in both groups. All our patients had a consistent improvement in ROM, both groups achieving a mean flexion of 90° at 2nd postoperative week and a mean flexion of 115° at 12th postoperative week. They recovered preoperatory levels of knee range of motion between 6 and 12th postoperative week. But the use of CPM in the post-TKA has remained controversial. Several studies have reported benefits of CPM during the acute phase [10, 20, 29, 30], while others report the absence of benefits [11, 26, 31]. But we should be careful on interpreting these different studies, because there is a high degree of heterogeneity in rehabilitation protocols, with different frequency and duration of CPM applied, anesthetic and analgesic drugs used, among other. In our study, we maintained the same fast recovery program, with the exception of the use and duration of CPM application. In the publication of Boese et al. [23] they compare three groups with different CPM application (full moving CPM, 90 degrees non-moving CPM, no CPM) at least two postoperative days, finding no differences between them in terms of ROM. In Joshi et al. [32] they used a similar rehabilitation program, but with a very different anesthesia protocol with peripheral nerve block option, and they found no benefits in CPM use. Richter et al. [33] compare two groups with more days of CPM application than us (10 post-op days), without differences in terms of ROM or clinical and functional results. Wirries et al. [34] showed same results at long-term follow-up, without clinical advantages in mobility and functional results.

In our study, we did not found repercussion of the CPM rehabilitation protocol to the SWA, except for hematoma. Our SWA analysis revealed a significantly increased number of patients in the no-CPM group with hematoma compared to the CPM group. This could be possible due to CPM may reduce swelling due to its pumping action, pushing blood and edema fluid away from the joint and periarticular tissues [5, 35]. McInnes et al. [27] and Montgomery et al. [36] observed a decrease in swelling with the use of CPM. However, we did not observe any difference in swelling in our SWA scores, according to other studies [5, 11, 23]. With respect to another SWA parameters, i.e., bleeding, Maniar et al. [5] concluded that CPM may tend to lead to a greater incidence of wound staining according to their classification. We did not see any such difference. Johnson et al. [37] pointed out that the transcutaneous oxygen tension on the lateral aspect of incision might decrease as the knee flexed more than 40°. In our patients, with more than 40° on flexion in the 1^st^ postoperative day, no skin necrosis was observed. Other studies suggest a positive benefit of CPM in biological tissues with respect to tissue healing, limb edema, hemarthrosis and knee function [10, 35, 38]. Our results are partly in agreement with these findings.

In terms of pain, the patients included in this study had a progressive reduction in pain during the course of follow-up, with no difference within groups. However, Denis et al. [17] showed that use of CPM might be beneficial for pain relief in the early postoperative stage. Cochrane review [20] find only low-quality evidence for the reduction in pain using CPM, with a mean reduction scores of 0.4 points (10 point scale) at 6th postoperative week, whereas in our study, we saw a difference within groups of only 0,1 points.

In addition, the Cochrane review [20] find that CPM reduced the possibility of performing a forced mobilization under anesthesia because of the stiffness after surgery. In our study, no patients required this kind of procedure in any of both groups.

The main strength of our study is one of the largest sample size studies in the literature, excluding metanalysis. Furthermore, the prospective and randomized controlled trial design strength the results. Nevertheless, this study has few limitations. The grouping of the participants to CPM used could not be blinded because of the nature of CPM device. In addition, some patients had undergone a previous TKA before this study, and thus knew that use of a CPM device was standard, potentially resulting in an effect that could produce an uncontrollable patient suggestion. SWA score, used for assess surgical wound aspect, even publicated it is not validated [25].

## Conclusions

The use of CPM under this protocol does not provide clinical improvement in terms of knee flexion, appearance of surgical wound or reduction pain after TKA, except for hematoma where we observed a decreased risk of postoperative using CPM.

## Data Availability

The datasets used and/or analyzed during the current study are available from the corresponding author on reasonable request.

## Abbreviations

CPM: Continuous passive motion
TKA: Total knee arthroplasty
SWA: Surgical wound aspect
SWAS: Surgical wound aspect score
OA: Osteoarthritis
ROM: Range of motion
SRP: Standardized rehabilitation program
